# Effect of the addition of β-mannanase on the performance, metabolizable energy, amino acid digestibility coefficients, and immune functions of broilers fed different nutritional levels

**DOI:** 10.3382/ps/pew076

**Published:** 2016-04-02

**Authors:** H. C. Ferreira, M. I. Hannas, L. F. T. Albino, H. S. Rostagno, R. Neme, B. D. Faria, M. L. Xavier, L. N. Rennó

**Affiliations:** * Department of Animal Science, Universidade Federal de Viçosa - UFV, Viçosa, Minas Gerais, Brazil; † Ilender Pharm. Corp., Campinas, São Paulo, Brazil

**Keywords:** β-mannanase, broiler, performance, metabolizable energy, immune function

## Abstract

Three experiments were conducted to evaluate the effect of β-mannanase **(BM)** supplementation on the performance, metabolizable energy, amino acid digestibility, and immune function of broilers. A total of 1,600 broilers were randomly distributed in a 4 × 2 factorial arrangement (4 nutritional levels × 0 or 500 g/ton BM), with 10 replicates and 20 broilers per pen. The same design was used in the energy and digestibility experiments with 8 and 6 replicates, respectively, and 6 broilers per pen. The nutritional levels **(**NL**)** were formulated to meet the nutritional requirements of broilers **(**NL1**)**; reductions of 100 kcal metabolizable energy **(**NL2**)**; 3% of the total amino acids (NL3); and 100 kcal metabolizable energy and 3% total amino acids (NL4) from NL1. The serum immunoglobulin (Ig) concentration was determined in two broilers per pen, and these broilers were slaughtered to determine the relative weight of spleen, thymus, and bursa of Fabricius. Throughout the experiment, the lower nutritional levels reduced (*P* < 0.05) body weight gain **(**BWG**)** and increased (*P* < 0.05) feed conversion **(**FCR**)** for the NL4 treatment. The BM increased (*P* < 0.05) the BWG values and improved (*P* < 0.05) the FCR of the broilers. The apparent metabolizable energy corrected for nitrogen balance (AMEn) values were reduced (*P* < 0.05) for NL2 and NL3. The BM increased (*P* < 0.05) the AMEn values and reduced (*P* < 0.05) the excreted nitrogen. NL3 and NL4 reduced (*P* < 0.05) the true ileal digestibility coefficients (TIDc) of the amino acids cystine and glycine, and BM increased (*P* < 0.05) the TIDc for all amino acids. The addition of BM reduced (*P* < 0.05) the relative weights of the spleen and bursa. NL2 increased (*P* < 0.05) the Ig values, whereas BM reduced (*P* < 0.05) the serum IgA, IgG, and IgM values of the broilers. This study indicates that using suboptimal nutrient levels leads to losses in production parameters, whereas BM-supplemented diets were effective in improving performance, energy values, and TIDc levels of amino acids and immune response of broilers.

## INTRODUCTION

Soluble non-starch polysaccharides **(SNSP)** are present in the hemicellulose fraction of fiber as primary cell wall constituents; they are also found in the secondary layer and include mannans, xylans, galactans, arabinans, and others (Dhawan and Kaur, 2007). This carbohydrate class is one of the main anti-nutritional factors in broiler diets. Monogastric animals cannot produce or produce insufficient amounts of the specific enzymes necessary to hydrolyze and digest such molecules (Brito et al., 2008).

The anti-nutritional properties of SNSP are caused by their solubility in liquid medium and increased viscosity, which interferes in the mixing of digestive enzymes with nutrients and prevents adequate digesta movement and product transport from the hydrolysis of molecules in the intestinal mucosa (Slominski, 2011). Gao et al. (2008) demonstrated that adding a carbohydrase (xylanase) to broiler feed at up to 21 d old can reduce the relative weights of the duodenum, jejunum, colon, and pancreas. This finding shows that the anti-nutritional effect is not only restricted to altering the digesta flow but also causes disturbances in the normal functioning of the digestive organs, with increased endogenous secretions such as water, proteins, electrolytes, and lipids (Angkanaporn et al., 1994).

The endocarbohydrase β-1,4-mannanase **(BM)** randomly cleaves the 1,4-β glycosidic bonds of the main mannan, galactomannan, glucomannan, and galactoglucomannan chains as well as the bonds of the mannan chain itself to yield mannobiose, mannotriose, and mannose as products during hydrolysis (Dhawan and Kaur, 2007). The use of BM in broiler diets is beneficial because it hydrolyzes a certain SNSP class, which reduces viscosity in the intestinal environment (Lee et al., 2003; Mehri et al., 2010); lower water retention from carbohydrate disaggregation into smaller molecules; and increases the availability of carbohydrates, which are absorbed and utilized by the animals (Saki et al., 2005). Moreover, BM supplementation can attenuate the immune response of broilers caused by the reduced production of immunoglobulins **(Ig)** (Li et al., 2010) and leukocytes (Mehri et al., 2010) because of the mannans present in the feed stimulate the immune system. Li et al. (2010) found that supplementing diets with β-mannanase reduced the weights of immunity-related organs such as the thymus and bursa of Fabricius.

Broiler diets are mainly based on corn and soybean meal, which are the main feed ingredients used in broiler production worldwide. Hsiao et al. (2006) analyzed 36 samples of soybean meal with husks from several countries and found that the mannan content of this feed ranged between 1.33 and 1.86%. The BM supplementation in monogastric animal feed improves performance, increases weight gain (Kim et al., 2003; Daskiran et al., 2004; Kong et al., 2011), and reduces feed conversion (Lee et al., 2003; Zou et al., 2006) because of the increased apparent metabolizable energy values (Daskiran et al., 2004; Kong et al., 2011; Mussini et al., 2011). In addition, increases are observed in the apparent amino acid digestibility coefficients (Mussini et al., 2011) when BM is added to feed.

Based on the observed benefits, BM may be employed as a strategy to reduce production costs because it can be used in lower energy broiler diets which leads to improved feed energy use, and the animals exhibit similar growth performance to broilers receiving adequate energy levels (Wu et al., 2005; Li et al., 2010). Three experiments were performed to evaluate the addition of BM to broiler diets with four different nutritional levels **(NL)** and determine the effects on performance, metabolizable energy, amino acid digestibility coefficients, relative immune organ weights, and serum Ig levels.

## MATERIALS AND METHODS

The experiments were performed in accordance with the animal experimentation ethical principles certified by the Livestock Use Ethics Committee (Comitê de Ética para o Uso de Animais de Produção - CEUAP) of the Universidade Federal de Viçosa. All of the experiments were conducted at the Poultry Farm of the Department of Animal Science, UFV, Brazil in April and May 2013.

### Enzymes

The enzymes used in all of the experiments were provided by the Ilender Pharmaceutical Corporation (Lima, Peru). Thermostable β-mannanase (CTCZYME) was produced from *Bacillus subtilis* fermentation and was included at a ratio of 500 g/ton according to the manufacturer's recommendations in a level of 800 units/g. One β-D-Mannanase unit is defined as the amount of enzyme which liberates 1 micromole of reducing sugar equivalent to D-mannose per minute at 50°C and pH 6.0.

### Experimental Design, Diets, and Animals

1,600 Cobb 500 male chicks at one-day-old were used in this trial. The experiment followed a completely randomized design in a 4 × 2 factorial arrangement, with 4 NL and 0 or 500 g/ton BM, in a total of 8 treatments and 10 replicates of 20 broilers per experimental unit (80 pens) housed in 1.0 m × 1.5 m pens, which were considered one experimental unit, and lined with wood shavings. The treatments without and with the addition of BM were as follows: control feed (**NL1** and NL1+BM), which was formulated to meet or exceed the broiler's requirements (Rostagno et al., 2011); reductions of 100 kcal metabolizable energy (**NL2** and NL2+BM); 3% of the total amino acids (**NL3** and NL3+BM); and 100 kcal metabolizable energy and 3% of the total amino acids (**NL4** and NN4+BM) from NL1 (Table 1). The mannan content present in the corn and soybean meal was determined by CBO – Análises Laboratoriais (Campinas, Brazil) by amperometric chromatography method. The experimental period was 6 weeks long (42 days) with an initial diet (0 to 3 weeks old) and final diet (3 to 6 weeks old) prepared for the different treatments.

**Table 1.** Ingredients and nutrient composition of the experimental diets (dry matter, DM = 89.41%).^1,2^

Ingredients (%)	Initial (0 – 3 weeks old)				Growth (3 – 6 weeks old)			
	NL1	NL2	NL3	NL4	NL1	NL2	NL3	NL4
Corn (0.27% mannans)	54.86	57.17	56.91	59.22	60.70	63.02	61.95	64.27
Soybean meal (0.69% mannans)	36.69	36.31	34.98	34.59	31.15	30.76	30.12	29.74
Soybean oil	4.111	2.181	3.765	1.835	4.447	2.517	4.244	2.314
Bicalcium phosphate	1.656	1.653	1.670	1.667	1.271	1.267	1.279	1.276
Limestone	1.018	1.020	1.018	1.021	0.848	0.851	0.848	0.851
Salt	0.483	0.482	0.483	0.482	0.452	0.451	0.452	0.451
Starch	0.300	0.300	0.300	0.300	0.320	0.320	0.320	0.320
DL-Methionine, 99%	0.277	0.275	0.265	0.263	0.238	0.236	0.223	0.220
L-Lysine HCl, 79%	0.162	0.169	0.168	0.175	0.163	0.170	0.154	0.161
L-Threonine, 98%	0.040	0.039	0.038	0.038	0.027	0.026	0.019	0.019
Mineral supplement^3^	0.110	0.110	0.110	0.110	0.100	0.100	0.100	0.100
Vitamin supplement^4^	0.110	0.110	0.110	0.110	0.100	0.100	0.100	0.100
Choline chloride, 60%	0.100	0.100	0.100	0.100	0.100	0.100	0.100	0.100
Salinomycin^5^, 12%	0.055	0.055	0.055	0.055	0.055	0.055	0.055	0.055
Avilamycin^6^, 10%	0.010	0.010	0.010	0.010	0.010	0.010	0.010	0.010
Antioxidant (BHT)	0.010	0.010	0.010	0.010	0.010	0.010	0.010	0.010
TOTAL	100	100	100	100	100	100	100	100
Calculated composition^7^								
Crude protein,%	21.25	21.26	20.63	20.64	19.17	19.18	18.78	18.79
Metabolizable energy (kcal/kg)	3,075	2,975	3,075	2,975	3,175	3,075	3,175	3,075
Calcium,%	0.894	0.894	0.894	0.894	0.724	0.724	0.724	0.724
Crude fiber,%	2.894	2.914	2.839	2.858	2.701	2.720	2.669	2.689
Mannans,%	0.401	0.405	0.395	0.399	0.378	0.382	0.375	0.379
Sodium,%	0.210	0.210	0.210	0.210	0.198	0.198	0.198	0.198
Fat,%	6.717	4.873	6.419	4.574	7.172	5.328	6.998	5.154
No phytic phosphorus,%	0.420	0.420	0.420	0.420	0.340	0.340	0.340	0.340
Digestible lysine,%	1.174	1.174	1.139	1.139	1.044	1.044	1.013	1.013
Digestible meth. + cysteine,%	0.846	0.846	0.821	0.821	0.762	0.762	0.739	0.739
Digestible arginine,%	1.349	1.345	1.302	1.298	1.194	1.189	1.165	1.161
Digestible threonine,%	0.763	0.763	0.740	0.740	0.679	0.679	0.659	0.659
Digestible tryptophan,%	0.240	0.240	0.231	0.230	0.211	0.210	0.206	0.204
Digestible valine,%.	0.904	0.904	0.877	0.877	0.814	0.814	0.798	0.798

^1^The diets were supplemented with 0 or 500 g/ton β-mannanase.

^2^NL1 = nutritional level 1(recommended levels according to Rostagno et al., 2011), NL2 = nutritional level 2 (reductions of 100 kcal metabolizable energy), NL3 = nutritional level 3 (reductions of 3% in the total amino acids), NL4 = nutritional level 4 (reductions of 100 kcal metabolizable energy and 3% in the total amino acids), BM = β-mannanase, BWG = body weight gain, FI = feed intake, FCR = feed conversion.

^3^Mineral supplement supplied per kg of feed: iron, 55.0 mg; copper, 11.0 mg; manganese, 77.0 mg; zinc, 71.5 mg; iodine, 1.10 mg; selenium, 0.330 mg.

^4^Vitamin supplement supplied per kg of feed: vitamin A, 8,250 IU; vitamin D3, 2,090 IU; vitamin E, 31 IU; vitamin B1, 2.20 mg; vitamin B2, 5.50 mg; vitamin B6, 3.08 mg; vitamin B12, 0.013 mg; pantothenic acid, 11.0 g; biotin, 0.077 mg; vitamin K3, 1.65 mg; folic acid, 0.77 mg; nicotinic acid, 33.0 mg.

^5^Coxistac: (12% sodium salinomycin);

^6^Surmax.

^7^The analyzed values of crude protein, total lysine, total meth. + cysteine, total arginine, total threonine, and total valine for the 0 to 3 weeks were 21.70, 21.07, 20.21, and 20.10%; 1.35, 1.27, 1.20, and 1.23%; 0.78, 0.79, 0.74, and 0.72%; 1.57, 1.59, 1.39, and 1.40; 0.93,0.89, 0.78, and 0.80; 1.02, 1.00, 0.92, and 0.91 for NL1, NL2, NL3, and NL4, respectively.

Light was continuously supplied, with 12 hours of natural light (6:00 AM to 6:00 PM) and 12 hours of artificial light (6:00 PM to 6:00 AM). The broilers had access to mash feed and water ad libitum.

The temperature in each experimental unit was maintained using 250-watt infrared lights (Empalux, Curitiba, Paraná, Brazil) until the broilers reached 14 days of age. After this period, the temperature was maintained using curtains.

For the trials to determine apparent metabolizable energy corrected for nitrogen balance **(AMEn)**, and true ileal digestibility coefficients **(TIDc)** of the amino acids, 800 Cobb 500 male chicks were reared in a brick barn inside fenced circles. They received corn and soybean meal based pre-starter feed, which meets the requirements proposed by Rostagno et al. (2011) and managed according to the breeder recommendation. 250-watt infrared lights (Empalux) were used to maintain the temperature within the circles. Mash feed and water were provided to the broilers ad libitum until the start of the experiments.

To determine the AMEn 384 chicks were transferred at 13 days of age to a room with 2 metal pens (30 cm wide × 65 cm long × 70 cm tall) that had compartments distributed into 2 levels and were arranged in a 68 m^2^ room with a ceiling height of approximately 2.8 m. Each compartment contained 24 cages each with a nipple and feeder. The room was heated with three 250-w infrared lights to maintain the temperature within the comfort zone. The broilers received feed and water ad libitum throughout the entire experimental period. The experiment followed a completely randomized design in a 4 × 2 factorial arrangement (4 NL and 0 or 500 g/ton BM), in a total of 8 treatments with 8 replicates and 6 broilers per pen. The treatments were the same as adopted in the performance trial corresponding to the 0- to 3-week-old stage.

The feed was provided ad libitum for 8 days, including 3 days for adaptation and 5 days for complete excreta collection from each experimental unit, with 12-hour intervals between each collection. Plastic-coated drip pans were placed under each pen.

To determine TIDc of amino acids, 360 chicks were transferred at 22 days of age to the same metal cages and kept under conditions identical to those of the previous experiment. The experiment followed a completely randomized design in a 4 × 2 factorial arrangement (4 NL and 0 or 500 g/ton BM), in a total of 8 treatments with 6 replicates and 6 broilers per pen. The treatments were the same as adopted in the performance trial corresponding to the 3- to 6-week-old stage. A protein-free diet **(PFD)** with 0 or 500 g/ton BM was formulated to determine the endogenous losses and calculate the TIDc of amino acids values (Table 2). PFD and PFD+BM treatments were used to determine the amino acid digestibility coefficients of the feeds without and with enzyme supplementation, respectively. As a source of acid insoluble ash **(AIA)**, 1% Celite (Imerys Minerales Arica LTDA, Chacalluta, Arica, Chile) was added to all treatments as indigestible marker to obtain the indigestibility factors **(IFs)**.

**Table 2.** Composition of the protein-free diet used to determine endogenous loss.^1^

Ingredients (%)	PFD
Starch	80.31
Sugar	5.00
Soybean oil	5.00
Dicalcium phosphate	2.10
Limestone	0.70
Salt	0.45
Potassium carbonate (K_2_CO_3_)	1.00
Corncob^2^	4.00
Mineral supplement^3^	0.08
Vitamin supplement^4^	0.15
Choline chloride, 60%	0.20
Antioxidant (BHT)	0.01
Acid-insoluble ash (Celite™)	1.00
Total	100.00
Crude protein,%^4^	0.176

^1^The protein-free diets (PFD) were supplemented with 0 or 500 g/ton β-mannanase enzyme.

^2^Considering the corncob with 4.4% of CP.

^3^Mineral supplement supplied per kg of feed: iron, 55.0 mg; copper, 11.0 mg; manganese, 77.0 mg; zinc, 71.5 mg; iodine, 1.10 mg; selenium, 0.330 mg.

^4^Vitamin supplement supplied per kg of feed: vitamin A, 8,250 IU; vitamin D3, 2,090 IU; vitamin E, 31 IU; vitamin B1, 2.20 mg; vitamin B2, 5.50 mg; vitamin B6, 3.08 mg; vitamin B12, 0.013 mg; pantothenic acid, 11.0 g; biotin, 0.077 mg; vitamin K3, 1.65 mg; folic acid, 0.77 mg; nicotinic acid, 33.0 mg.

### Performance, AMEn, Nitrogen Balance, TIDc, and Immune Function

The barn temperature was measured daily (at 4:00 PM). The minimum and maximum temperatures average were as follows: 19.2°C and 32.0°C for 0 to 7 days of age, 20.5 ± 1°C and 28.0 ± 1.5°C for 8 to 21 days of age, and 18.3 ± 2°C and 27.1 ± 1.3°C for 21 to 42 days of age.

At 1, 21, and 42 days old, the broiler weight, feed supply, and feed scraps were recorded for each experimental unit to determine the body weight gain **(BWG)**, average feed intake **(FI)** and feed conversion **(FCR)** during the 0 to 3, 3 to 6, and 0 to 6 week periods.

In the energy trial, excreta from each cage were weighed at the end of the experimental period and homogenized, and 300 g samples were removed, pre-dried at 65°C for 72 hours, and ground in a ball mill (Tecnal Equipamentos para Laboratório, TE-350, São Paulo, Brazil) for 5 minutes to a fine mixture.

In the digestibility trial, the broilers were slaughtered to collect the ileal digesta after the 5-day adaptation period to the experimental diets. The abdominal cavity of the broilers was opened, and all of the intestinal content present at 40 cm from the terminal ileum anterior to ileocecal junction was removed. The ileal digesta from the broilers of each replicate were combined to form the composite sample for each treatment. The broilers were constantly stimulated to consume feed before slaughter to avoid empty digestive tracts, which would compromise the collection procedure. The ileal digesta samples were freeze-dried at −40°C for 72 hours, and 50 g subsamples were collected.

At 42 days of age, two broilers per replicate were randomly selected, and a blood sample was collected by puncturing the vena cava using 6 mL vacuum blood collection tubes with heparin as an anticoagulant (Labor Import, São Paulo, Brazil) and then centrifuged (Fanem, model 206-R, Tecnal Equipamentos para Laboratório 10000, São Paulo, Brazil) at 10,000 rpm for 15 minutes (2,260 × *g*). The obtained serum was pipetted and maintained in 2 mL Eppendorf tubes at −20°C.

The same broilers that had blood collected were weighed and slaughtered at 42 days of age. After slaughter, a longitudinal cut was made on the abdominal cavity to collect the bursa of Fabricius and spleen, and another longitudinal cut was made on the neck to remove the thymus. The organs were then weighed to determine the immune organ relative weight.

### Chemical Analyses

The diets and excreta were analyzed (AOAC, 1990) for dry matter **(DM)** and crude protein **(CP)** at the Laboratory of Animal Nutrition of the Department of Animal Science, UFV, Brazil. The Kjeldahl method was used to determine the nitrogen in the diets and feces according to official analysis methods (AOAC, 1990). The excreted nitrogen **(EN)** was calculated by multiplying the total amount excreted in DM by the percentage of nitrogen in the excreta also in DM. The same was applied to the consumed nitrogen **(CN)** calculation. The nitrogen retained **(RN)** was calculated by subtracting EN from the CN. The Nitrogen retained in percent **(%RN)** was calculated in relation the amount of nitrogen consumed. The nitrogen balance **(NB)** was obtained from the amount of consumed nitrogen minus excreted nitrogen, and the gross energy **(GE)** values were determined using a C5001 adiabatic calorimetric pump (IKA-Werke GmbH & Co. KG, Staufen, Germany). The AMEn values were calculated based on the GE values of the feeds and feces using the equation described by Matterson (1965).

The DM analyses (AOAC, 1990) of the diets and ileal digesta collected from the broiler were performed for the digestibility calculations. The laboratory analyses to determine the amino acid content of the diets and excreta were performed by CBO – Análises Laboratoriais (Campinas, São Paulo, Brazil) using HPLC (high performance liquid chromatography). The AIA was analyzed using the technique described by Van Keulen and Young (1977) to determine the TIDc of amino acids.

The serum levels of IgA, IgG, and IgM were obtained by immunoturbidimetric assay using kits (Bioclin, Belo Horizonte, Minas Gerais, Brazil) following the manufacturer's instructions using a BS 200E apparatus (Mindray, Nanshan, China).

The relative weight of each immune organ (spleen, thymus, and bursa) was calculated according to the equation: relative immune organ weight = immune organ weight/BW.

### Statistical Analysis

The data were analyzed using a completely randomized design in a 4 × 2 factorial arrangement by analysis of variance (ANOVA) using the PROC GLM procedure (general linear model) with SAS software (SAS Institute, 1990). The “slice” function was used to reveal significant interactions, and Tukey's test at the P ≤ 0.05 significance level was used to identify significant differences.

## RESULTS

### Performance

Interactions were not observed (*P* > 0.05) among the four nutritional levels without or with BM for all of the periods analyzed (Table 3). At 21 days old, the broilers fed the NL1 diet exhibited higher BWG (*P* < 0.05) compared with those that received diets with lower nutritional levels NL2 and NL4, which showed reduced BWG by 3.46 and 5.31%, respectively. The reduced BWG of the broilers was reflected in increased FCR, with the broilers in the NL2 and NL4 treatments exhibiting FCR values that were 3.52 and 5.52% higher (*P* < 0.05) compared with those in the NL1 treatment. However, there was no difference in BWG (*P* < 0.05) and FCR (*P* > 0.05) between the broilers in the NL1 and NL3 treatments. BM supplementation did not affect (*P* > 0.05) performance at the 1 to 21 days old stage. The FI was unaffected (*P* > 0.05) by the NL treatment or enzyme addition.

**Table 3.** Effect of different nutritional levels and β-mannanase supplementation on broiler performance.^1,2^

	Nutritional level				BM			Sources of variation		
	NL1	NL2	NL3	NL4	0 g/ton	500 g/ton	SEM	NL	BM	NL*BM
0 to 3 weeks old										
BWG (g)	867^x^	837^y,z^	847^x,y^	821^z^	839	847	0.029	0.001	0.22	0.46
FI (g)	1,190	1,186	1,180	1,190	1,186	1,188	0.037	0.82	0.85	0.65
FCR	1.37^x^	1.42^y^	1.39^x,y^	1.45^z^	1.42	1.40	0.037	0.001	0.15	0.72
3 to 6 weeks old										
BWG (g)	1,815^x^	1,782^x,y^	1,768^x,y^	1,731^y^	1,745^Y^	1,803^X^	0.062	0.001	0.001	0.81
FI (g)	3,001	2,982	2,961	2,941	2,954^Y^	2,989^X^	0.075	0.73	0.039	0.87
FCR	1.65^x^	1.68^x,y^	1.67^x,y^	1.71^y^	1.70^Y^	1.66^X^	0.059	0.011	0.005	0.67
Entire period										
BWG (g)	2,683^x^	2,618^y^	2,613^y^	2,549^z^	2,583^Y^	2,648^X^	0.073	0.001	0.001	0.53
FI (g)	4191	4168	4150	4120	4139	4175	0.095	0.12	0.094	0.80
FCR	1.56^x^	1.59^x^	1.58^x^	1.63^y^	1.60^Y^	1.57^X^	0.040	0.001	0.005	0.60

^x–z^Means followed by different uppercase letters in the same row differ according to the Tukey test (*P* < 0.05).

^X,Y^Means followed by different uppercase letters in the same row differ according to the F test (*P* < 0.05).

^1^Mean observations were calculated using 10 replicates, where the pen constituted an experimental unit.

^2^NL1 = nutritional level 1 (recommended levels according to Rostagno et al., 2011), NL2 = nutritional level 2 (reductions of 100 kcal metabolizable energy), NL3 = nutritional level 3 (reductions of 3% in the total amino acids), NL4 = nutritional level 4 (reductions of 100 kcal metabolizable energy and 3% in the total amino acids), BM = β-mannanase, BWG = body weight gain, FI = feed intake, FCR = feed conversion.

In the 22 to 42 d-old period, the broilers in the NL1 treatment exhibited higher BWG (*P* < 0.05) compared with the broilers in the NL4 treatments, which 4.63% lower, respectively. The FI was unaffected (*P* > 0.05) by lower nutritional levels. The broilers with BM-supplemented feed exhibited higher BWG and FI (*P* < 0.05) and lower FCR (*P* < 0.05) compared with those that were not fed a supplemented diet, in the proportions of 3.32%, 1.17%, and 2.40%, respectively.

Throughout the entire experimental period (1 to 42 days of age), the broilers in the NL1 treatment exhibited higher BWG (*P* < 0.05) compared with those that received diets with lower NL. Reductions in NL decreasing the BWG of the animals by 2.42, 2.61, and 4.62% for NL2, NL3, and NL4, respectively, compared with NL1. The FCR increased (*P* < 0.05) in the broilers in the NL4 treatment compared with those from the other treatments, and the values were 4.30% higher than in the NL1 treatment. Although the broilers in the NL1 treatment exhibited higher BWG, their FCR was similar to those in the NL2 and NL3 treatments. The BM supplementation in the diet increased the BWG (*P* < 0.05) by 2.45% and improved the FCR (*P* < 0.05) by 1.87% compared with diets that did not receive enzyme supplements. There was no difference in FI (*P* > 0.05) throughout the entire experimental period when the treatments with different nutritional levels and BM supplements were analyzed.

### Apparent Metabolizable Energy Corrected for Nitrogen Balance (AMEn), Consumed Nitrogen (CN), Excreted Nitrogen (EN), Retained Nitrogen (RN), and Percentage Retained Nitrogen (%RN)

Interactions were not observed (*P* > 0.05) between NL and the BM supplementation for the AMEn, and EN values (Table 4). The 100 kcal less energy and 100 kcal less energy + 3% less total amino acid treatments reduced the AMEn (*P* < 0.05) by 1.37 and 1.72% for NL2 and NL4, respectively, compared with NL1. NL1 and NL3 exhibited similar AMEn values. The EN was unaffected by lowering the NL (*P* > 0.05).

**Table 4.** Effect of different nutritional levels and β-mannanase supplementation on energy values of apparent metabolizable energy (AME), apparent metabolizable energy corrected for nitrogen balance (AMEn), consumed nitrogen (CN), excreted nitrogen (EN), retained nitrogen (RN), and percentage retained nitrogen (%RN).^1,2^

		Nutritional levels						Sources of variation		
	BM	NL1	NL2	NL3	NL4	Mean	SEM	NL	BM	NL*BM
AMEn	0	3,186	3,135	3,212	3,134	3,167^Y^	38.598	0.001	0.001	0.77
	500	3,238	3,199	3,245	3,179	3,215^X^				
Mean		3,211^x^	3,167^y^	3,228^x^	3,156^y^					
CN	0	3.35^a,A^	2.96^b,c,A^	2.80^c,B^	3.06^b,A^	3.04	0.140	0.001	0.001	0.024
	500	3.31^a,A^	3.21^a,A^	3.16^a,A^	3.10^a,A^	3.20				
Mean		3.34	3.09	2.99	3.08					
EN	0	0.85	0.95	0.91	0.96	0.92^Y^	0.083	0.27	0.007	0.14
	500	0.88	0.86	0.78	0.88	0.85^X^				
Mean		0.86	0.89	0.85	0.91					
RN	0	2.48^a,A^	2.00^b,c,B^	1.86^c,B^	2.11^b,A^	2.12	0.114	0.001	0.001	0.001
	500	2.48^a,A^	2.40^a,A^	2.41^a,A^	2.18^b,A^	2.37				
Mean		2.48	2.20	2.13	2.16					
%RN	0	75.0^a,A^	68.1^b,B^	66.8^b,B^	69.2^b,A^	69.81	2.391	0.001	0.001	0.001
	500	74.0^a,A^	74.1^a,A^	75.2^a,A^	71.2^a,A^	73.68				
Mean		74.53	71.16	71.04	70.25					

^x–z^Means followed by different uppercase letters in the same row differ according to the Tukey test (*P* < 0.05).

^X,Y^Means followed by different uppercase letters in the same row differ according to the F test (*P* < 0.05).

^a–c^Means followed by different uppercase letters in the same row differ according to nutritional levels.

^A,B^Means followed by different uppercase letters in the same row differ according to enzyme supplementation.

^1^Mean observations were calculated using 10 replicates, where the pen constituted an experimental unit.

^2^NL1 = nutritional level 1 (recommended levels according to Rostagno et al., 2011), NL2 = nutritional level 2 (reductions of 100 kcal metabolizable energy), NL3 = nutritional level 3 (reductions of 3% in the total amino acids), NL4 = nutritional level 4 (reductions of 100 kcal metabolizable energy and 3% in the total amino acids), BM = β-mannanase, AMEn = apparent metabolizable energy for nitrogen balance (kcal/kg), CN = consumed nitrogen (g/broiler/day), EN = excreted nitrogen (g/broiler/day), RN = retained nitrogen (g/broiler/day), and%RN = percentage retained nitrogen (%).

The BM supplementation increased (*P* < 0.05) AMEn by 1.49% compared with the broilers fed the treatments without the enzyme addiction. The EN was reduced (*P* < 0.05) with enzymatic supplementation, with values that were 2.20% lower than treatments without supplementation.

Significant interactions (*P* < 0.05) were observed for CN, RN, and %RN among the nutritional levels and BM addiction. The lower nutritional levels resulted in reduced CN, RN, and %RN values (*P* < 0.05), whereas BM supplementation recovered these values in the NL2 and NL3 treatments, which presented similar values to those of the NL1 treatment (*P* < 0.05). The NL1 and NL4 treatments remained unaffected (*P* > 0.05) by enzymatic supplementation.

### True Ileal Digestibility Coefficient of Amino Acids

Interactions were not observed (*P* > 0.05) between the lower nutritional levels and BM supplementation on the TIDc of amino acids (Table 5). The 3% less total amino acids treatment reduced the TIDc (*P* < 0.05) of cystine and glycine for the NL3 and NL4 by 3.57% and 8.49% and 3.19% and 4.88%, respectively, compared with NL1. The TIDc of lysine, valine, and proline decreased (*P* < 0.05) only in the NL4 treatment, with values of 1.02%, 1.40%, and 1.21%, respectively.

**Table 5.** Effect of different nutritional levels and β-mannanase supplementation on the true ileal digestibility coefficients (TIDc) values and crude protein (CP) percentage data.^1,2^

	Nutritional levels				BM			Sources of variation		
	NL1	NL2	NL3	NL4	0 g/ton	500 g/ton	SEM	NL	BM	NL*BM
Lysine	88.6^x^	88.5^x,y^	88.2^x,y^	87.7^y^	87.8^Y^	88.7^X^	0.739	0.027	0.001	0.92
Methionine	92.0^y^	92.6^x,y^	93.3^x^	92.6^x,y^	92.1^Y^	93.2^X^	0837	0.008	0.001	0.25
Cystine	75.6^x^	74.9^x,y^	73.0^y^	69.7^z^	72.6^Y^	74.0^X^	2.310	0.001	0.032	0.18
Threonine	83.4	82.9	82.6	81.8	81.8^Y^	83.5^X^	1.981	0.27	0.005	0.85
Arginine	89.7	90.0	89.6	89.5	89.1^Y^	90.2^X^	0.745	0.41	0.001	0.84
Glycine	78.0^x^	77.7^x^	75.6^y^	74.3^y^	75.3^Y^	77.5^X^	1.809	0.001	0.001	0.23
Serine	83.3	82.6	82.6	82.1	81.9^Y^	83.4^X^	1.586	0.37	0.003	0.84
Valine	85.5^x,y^	85.8^x^	86.1^x^	84.4^y^	85.1^Y^	85.8^X^	1.182	0.003	0.037	0.15
Isoleucine	85.1	85.0	85.8	85.2	84.9^Y^	85.7^X^	1.054	0.31	0.016	0.45
Leucine	84.4	84.2	84.5	83.9	83.7^Y^	84.8^X^	1.150	0.58	0.002	0.86
Histidine	85.6	84.5	85.0	84.7	84.4^Y^	85.5^X^	1.125	0.10	0.001	0.97
Phenylalanine	86.1	86.1	86.3	86.3	85.6^Y^	86.8^X^	1.111	0.94	0.001	0.92
Tyrosine	85.4	84.6	85.0	85.4	84.4^Y^	85.9^X^	1.166	0.27	0.001	0.49
Alanine	84.3	84.1	85.2	85.1	83.8^Y^	85.5^X^	1.201	0.063	0.001	0.70
Proline	81.9^x^	81.1^x,y^	80.9^x,y^	79.6^y^	80.1^Y^	81.7^X^	1.477	0.004	0.001	0.74
Aspartic	90.4	89.7	89.3	90.5	89.2^Y^	90.8^X^	1.753	0.41	0.004	0.17
Glutamic	89.8	89.7	89.4	89.9	89.1^Y^	90.3^X^	1.216	0.79	0.002	0.79
CP	86.5	85.4	86.5	85.6	85.1^Y^	86.9^X^	1.295	0.080	0.001	0.95

^x–z^Means followed by different uppercase letters in the same row differ according to the Tukey test (*P* < 0.05).

^X,Y^Means followed by different uppercase letters in the same row differ according to the F test (*P* < 0.05).

^1^Mean observations were calculated using 10 replicates, where the pen constituted an experimental unit.

^2^NL1 = nutritional level 1 (recommended levels according to Rostagno et al., 2011), NL2 = nutritional level 2 (reductions of 100 kcal metabolizable energy), NL3 = nutritional level 3 (reductions of 3% in the total amino acids), NL4 = nutritional level 4 (reductions of 100 kcal metabolizable energy and 3% in the total amino acids), BM = β-mannanase, TIDc = true ileal digestibility coefficient, and CP = crude protein.

BM supplementation increased the TIDc (*P* < 0.05) of all of the amino acids as follows: 1.01% for lysine, 1.20% for methionine, 2.02% for cystine, 2.06% for threonine, 1.28% for arginine, 2.97% for glycine, 1.78% for serine, 0.87% for valine, 0.91% for isoleucine, 1.32% for leucine, 1.36% for histidine, 1.34% for phenylalanine, 1.78% for tyrosine, 2.04% for alanine, 2.07% for proline, 1.72% for aspartic, and 1.31% for glutamic. In addition, the TIDc of crude protein increased (*P* < 0.05) by 2.04%.

### Immune Function

Interactions were not observed (*P* > 0.05) between the NL and BM supplementation treatments for the bursa of Fabricius and spleen, whereas an interaction was observed (*P* < 0.05) for the relative weight of the thymus (Table 6). Immune organ relative weights were unaffected (*P* > 0.05) by reduced nutritional levels. BM supplementation reduced (*P* < 0.05) the immune organ relative weights in the broilers fed the supplemented diet compared with those that did not receive the enzyme. The weights of the bursa of Fabricius and spleen decreased by 11.8% and 13.4%, respectively, with BM supplementation. In addition, BM supplementation produced a greater reduction (*P* < 0.05) in the relative weight of the thymus (14.06%) compared with those of the non-supplemented diets, although the relative weight of the thymus decreased in the NL1 treatment group (*P* < 0.05) only. Enzyme addition did not have an effect (*P* > 0.05) on the weight of this organ for the other NL.

**Table 6.** Effect of different nutritional levels and β-mannanase supplementation on immune organ relative weight and serum immunoglobulin concentration of 42-day-old broilers.

		Nutritional levels						Sources of variation		
	BM	NL1	NL2	NL3	NL4	Mean	SEM	NL	BM	NL*BM
Immune organ relative weight (g/kg)										
Bursa	0	1.86	2.05	1.88	1.99	1.95^Y^	0.389	0.83	0.005	0.62
	500	1.69	1.71	1.79	1.67	1.72^X^				
Mean		1.78	1.88	1.84	1.83					
Spleen	0	1.06	1.12	1.05	0.91	1.04^Y^	0.202	0.22	0.001	0.53
	500	0.89	0.91	0.90	0.87	0.90^X^				
Mean		0.98	1.01	0.98	0.90					
Thymus	0	3.59^a,A^	3.00^a,A^	3.06^a,A^	3.12^a,A^	3.20	0.701	0.94	0.003	0.005
	500	2.23^a,B^	2.97^a,A^	2.86^a,A^	2.94^a,A^	2.75				
Mean		2.91	2.99	2.97	3.03					
Immunoglobulin (mg/dL)										
IgA	0	5.90^b,A^	9.35^a,A^	5.25^b,A^	5.25^b,A^	6.44	1.736	0.001	0.001	0.001
	500	4.85^a,A^	4.75^a,B^	3.30^a,B^	4.25^a,A^	4.28				
Mean		5.37	7.05	4.27	4.75					
IgG	0	1.70^b,A^	3.05^a,A^	1.85^b,A^	2.20^b,A^	2.20	0.785	0.001	0.001	0.003
	500	1.45^a,A^	1.55^a,B^	1.20^a,A^	1.10^a,B^	1.33				
Mean		1.58	2.30	1.53	1.65					
IgM	0	18.1^b,A^	29.3^a,A^	18.0^b,A^	19.30^b,A^	21.2	4.246	0.001	0.001	0.001
	500	15.8^a,A^	14.9^a,B^	9.80^b,B^	13.35^a,b,B^	13.4				
Mean		16.9	22.1	13.9	16.3					

^x–z^Means followed by different uppercase letters in the same row differ according to the Tukey test (*P* < 0.05).

^X,Y^Means followed by different uppercase letters in the same row differ according to the F test (*P* < 0.05).

^a–c^Means followed by different uppercase letters in the same row differ according to nutritional levels.

^A,B^Means followed by different uppercase letters in the same row differ according to enzyme supplementation.

^1^Mean observations were calculated using 10 replicates, where the pen constituted an experimental unit.

^2^NL1 = nutritional level 1 (recommended levels according to Rostagno et al., 2011), NL2 = nutritional level 2 (reductions of 100 kcal metabolizable energy), NL3 = nutritional level 3 (reductions of 3% in the total amino acids), NL4 = nutritional level 4 (reductions of 100 kcal metabolizable energy and 3% in the total amino acids), BM = β-mannanase, IgA = immunoglobulin A, IgG = immunoglobulin G, and IgM = immunoglobulin M.

Significant interactions were observed (*P* < 0.05) between the NL treatments and BM supplementation for the IgA, IgG, and IgM values. The NL2 exhibited the highest (*P* < 0.05) IgA value, which was 23.83% higher than in the NL1 and 39.43% and 32.62% higher than in the NL3 and NL4. In addition, BM supplementation reduced (*P* < 0.05) the serum IgA concentrations of the broilers by 33.54%, but did not influence (*P* > 0.05) the IgA values in the NL1 and NL4 treatments.

The NL2 increased the IgG concentration (*P* < 0.05) in the broilers by 31.30%, 33.48%, and 28.26% compared with the NL1, NL3, and NL4 treatments, respectively. The others NL remained similar (*P* > 0.05). The BM supplementation reduced (*P* < 0.05) the IgG value of the broilers by 39.54% compared with those that did not receive the supplementation. However, BM did not reduce the IgG values (*P* > 0.05) in the NL1 and NL3 treatments, although it did have an effect on the IgG the values (*P* < 0.05) in the NL2 and NL4 treatments.

The NL2 treatment increased (*P* < 0.05) the IgM values by 23.29% and 26.32% compared with NL1 and NL4 treatments, respectively, which remained similar by 37.25% compared with NL3. The BM supplementation reduced the IgM values (*P* < 0.05) of the broilers by 36.46% compared with those that did not receive supplementation. The addiction of enzyme reduced the IgM values (*P* < 0.05) in the NL2, NL3, and NL4 treatments but did not affect the NL1 treatment (*P* > 0.05).

## DISCUSSION

The results of the present study showed that lower nutritional levels can compromise broiler performance regardless of the stage analyzed. The BWG reduced with decreasing nutritional levels, with the broilers fed the reduced energy and reduced amino acids diet the most compromised. Even with lower BWG, the nutritional levels at 100 kcal less metabolizable energy or 3% less total amino acids successfully maintained FCR values similar to that of the NL1 treatment. Broilers fed reduced energy and reduced amino acid diets exhibiting the worst results, thus revealing the additive effect of 100 kcal less metabolizable energy + 3% less total amino acids. These results corroborate with Li et al. (2010) which was observed decrease average daily gain (**ADG**) and increased FCR in broilers fed with low-energy diets without enzymatic supplementation.

The BM supplementation did not improve performance during the first stage, and the results of this study corroborate those of Li et al. (2010), Mehri et al. (2010), and Zou et al. (2006), who did not observe improved broiler growth until the animals reached 3 weeks of age. This may have been caused by the physiologically immature state of the animals preventing them from benefiting from the enzymatic supplement or the animals being close to their genetic potential. However, BM supplementation increased the BWG and FI values and improved the FCR during the 4- to 6-week-old stage and throughout the entire experiment. The present study corroborates Zou et al. (2006), who found improved ADG and FCR for the same stages when including 0.025% and 0.05% BM in corn- and soybean-meal-based feed. Jackson et al. (2004) worked with feed devoid of growth promoters and found that supplementing 80 million U/ton BM promoted increased ADG in the broilers, and supplementing 110 million U/ton BM exerted an additive effect on increased ADG and improved FCR in the broilers compared with the control treatment. Additional studies reinforce the function of BM in improving broiler performance (Lee et al., 2003; Daskiran et al., 2004; Li et al., 2010; Cho and Kim, 2013). This improvement is caused by of a series of benefits derived from BM supplementation, such as hydrolysis of a mannans class considered one of the main anti-nutritional factors of broiler diets. The reduction in the size of these molecules by the enzyme action allows improves the digestibility of nutrients such as dry matter (Cho and Kim, 2013), crude protein, and crude fiber (Li et al., 2010) and produces higher metabolizable energy values (Daskiran et al., 2004; Kong et al., 2011) and increased ileal digestibility coefficients of amino acids (Mussini et al., 2011).

The nutritional levels with 100 kcal less metabolizable energy exhibited reduced AMEn values compared with the control level and diets with only 3% less total amino acids. These results confirm that formulating diets with suboptimal nutritional energy levels was responsible for the lower energy supply to the broilers. The BM supplementation increased the AMEn values compared with diets that did not receive the supplement. Mussini et al. (2011) observed an increase of 41.62% in the ileal apparent metabolizable energy of broiler fed diets based on corn and soybean meal and with 0.1% BM supplementation. Increased energy values were also observed by Kong et al. (2011) when using a diet with recommended nutritional levels and another with 100 kcal less energy with and without BM supplementation. The AMEn increased by 4.96% for broiler chickens supplemented with BM. This increase can be explained though BM supplementation may have increased the hydrolysis of SNSP into smaller molecules. This allows higher energy input because mannose, one of the residues produced by this process, can be absorbed by the animal (Saki et al., 2005) and used as an energy source. Another hypothesis was addressed by Meng et al. (2005) in an in vitro study on the activity of carbohydrase preparations on non-starch polysaccharides. Degradation by BM produces oligosaccharides and free sugars, thus forming potential fermentation substrates for bacterial cells present in the broilers’ intestine. Once fermented, volatile fatty acids are released that can be used by the broilers, thus increasing the AMEn values in corn- and soybean-meal-based feed. In addition, studies have shown that using BM can reduce the size of viscera related to the digestion process (Lee et al., 2003; Li et al., 2010). Therefore, the size of the gastrointestinal tract contributes to the broilers’ basal metabolism, and reductions in size help to reduce the broilers’ heat production, which promotes an increase in dietary energy values.

A release of 48 kcal AMEn for chickens fed with BM enzyme was observed. Enzyme supplementation allows a reduction in nutrient levels of diets without losses in animal performance. Also, with the release of energy, the inclusion of energetic ingredients can be decreased, which will reflect in reduction of production costs.

In this study, BM supplementation reduced the EN of the broilers. This finding shows the importance of using the enzyme to improve the broilers’ nutrient use because less nitrogen was eliminated in the environment, thus reducing the environmental impacts of broiler production. The BM enzyme was not able to increase NC, RN, and R% values of animals that received NL4. It was suggested that this characteristic is due to the additive effect of reduction of 100 kcal less metabolizable energy + 3% less total amino acids in NL4, which may have generated a bigger challenge for animals and even the enzyme supplementation failed to recover these values.

The nutritional levels with 3% less total amino acids and 100 kcal less energy + 3% less total amino acids produced a reduction in the TIDc of cystine and glycine as well as reduced coefficients for lysine, valine, and proline. Therefore, the reduced nutritional levels compromised the TIDc of the amino acids, which were used at suboptimal levels or levels below those required by the animal. The results corroborate other studies showing that mixtures of carbohydrases (Kim et al., 2003) or enzymatic complexes containing carbohydrase and protease (Romero et al., 2013) can increase the apparent amino acid digestibility coefficient values for swine and broilers, respectively. Selle et al. (2009) observed an increase in the apparent digestibility coefficient of most amino acids with xylanase supplementation compared with the control treatment for wheat-based broiler feed. Mussini et al. (2011) observed a linear increase in the apparent digestibility coefficients of lysine, methionine, threonine, tryptophan, arginine, leucine, isoleucine, cystine, and valine with increased BM levels in broiler diets of 0, 0.025, 0.050, and 0.1%. The SNSP can increase the endogenous excretion of amino acids (Angkanaporn et al., 1994) with increased ileal digesta viscosity (Lee et al., 2003), which leads to a longer retention time, and they stimulate the endogenous secretion of amino acids and increase mucin production by caliciform cells present at the brush border (Selle et al., 2009), compromising their digestibility. Thus, the improved TIDc found in the present study may be explained by a decrease in the ileal digesta viscosity and amount of caliciform cells (Mehri et al., 2010) because of the increased hydrolysis of SNSP with BM supplementation, which allows for greater contact between the enzyme and substrate and facilitates protein and amino acid digestion. In addition, mucin production is reduced through improved fiber digestion (Romero et al., 2013), and less amino acids are lost during the production.

In this study, reduced nutritional levels did not affect the relative weight of the immune organs, whereas BM supplementation reduced the weight of the bursa of Fabricius and spleen. Diets with nutritional deficiency provided in this experiment promoted greater stimulation of the immune system and BM supplementation was not able to attenuate the thymus’ weight. The relative weight of this organ was decreased only in animals receiving the NL1. Similar results were found by Li et al. (2010), where the relative weight of the thymus and bursa decreased at 3 weeks of age, and the relative weight of the bursa decreased at 6 weeks of age in broilers that received 1 and 2 g/kg of BM supplementation. Animals with activated immune systems can redirect nutrients acquired in the diet to produce immune response-related molecules, which reduce the nutrients required for maintenance and growth and are directly reflected in performance losses.

An analysis of the serum Ig concentration of the broilers showed that the 100 kcal energy reduction treatment promoted greater increases in the values of the three analyzed Ig types compared with the other treatments, with subsequent decreases in IgA and IgM for the NL3 treatment. These results suggest that the reduced energy supply promoted higher metabolic stress in the broilers compared with the other reduced levels; thus, energy deficiency represented a greater immune system stimulus. The reduced IgM values in diets with 3% less total amino acids and with BM supplementation may be explained by deficient amounts of amino acids for the formation and production of immune response-related proteins. Glick et al. (1981) used red blood cell injections from goat blood as an antigen and administered it to broiler chickens fed protein- or energy-deficient diets or diets deficient in both proteins and energy, and they observed lower IgG values at both levels compared with the broilers fed a control diet. The deficiency in arginine in diets with 3% less total amino acids may have promoted reductions in IgA and IgM values because supplementing arginine in diets with reduced protein can increase the amount of broiler antibodies against the virus that causes Newcastle disease (Jahanian, 2009). Moreover, BM supplementation reduced the IgA, IgG, and IgM values in broiler serum. When found in the intestinal lumen, the mannans are potent stimulators of the immune system. The BM enzyme acts in reducing the mannans content in the intestinal tract resulting in a lower stimulation of the immune system. The present study corroborates Li et al. (2010), who found reduced IgG and IgM values in 3-week-old broilers fed 1 and 2 g/kg of BM. Additionally, BM supplementation can reduce the concentration of heterophils and lymphocytes in 35-day-old broilers (Mehri et al., 2010). Thus, it is clear that SNSP are potential immune system stimulators for broilers, and BM supplementation can reduce the stress caused by this carbohydrate class.

When we observe the interactions, the BM enzyme successfully reduces the immunoglobulins concentrations in the serum of broilers fed with different nutritional plans, except for birds fed with NL. This shows that the BM action is effective only when the animals are under physiological stress, in this case, nutritional deficiency of their diets.

This study clearly showed that diets formulated below the animals’ nutritional demand can lead to losses in performance, AMEn levels, and amino acid TIDc. There are advantages of using BM in diets because it increased BWG and improved FCR throughout the entire experimental period. In addition, increased AMEn, amino acid TIDc, and immune system attenuation were observed; thus, BM supplementation is an important tool for formulating diets to improve performance parameters.
